# High usage of medial unicompartmental knee arthroplasty negatively influences total knee arthroplasty revision rate

**DOI:** 10.1007/s00167-021-06650-4

**Published:** 2021-06-30

**Authors:** Antonio Klasan, Mei Lin Tay, Chris Frampton, Simon William Young

**Affiliations:** 1 Department for Orthopaedics and Traumatology, Kepler University Hospital GmbH, Krankenhausstrasse 9, 4020 Linz, Austria; 2 Johannes Kepler University Linz, Altenberger Strasse 69, 4040 Linz, Austria; 3 North Shore Hospital, 124 Shakespeare Road, Takapuna, 0620 Auckland, New Zealand; 4 University of Auckland, CBD 1010, Auckland, New Zealand; 5 University of Otago, 4 Oxford Terrace, 8011 Christchurch, New Zealand

**Keywords:** Unicompartmental knee arthroplasty, Total knee arthroplasty, Survivorship, Revision

## Abstract

**Purpose:**

Surgeons with higher medial unicompartmental knee arthroplasty (UKA) usage have lower UKA revision rates. However, an increase in UKA usage may cause a decrease of total knee arthroplasty (TKA) usage. The purpose of this study was to investigate the influence of UKA usage on revision rates and patient-reported outcomes (PROMs) of UKA, TKA, and combined UKA + TKA results.

**Methods:**

Using the New Zealand Registry Database, surgeons were divided into six groups based on their medial UKA usage: < 1%, 1–5%, 5–10%, 10–20%, 20–30% and > 30%. A comparison of UKA, TKA and UKA + TKA revision rates and PROMs using the Oxford Knee Score (OKS) was performed.

**Results:**

A total of 91,895 knee arthroplasties were identified, of which 8,271 were UKA (9.0%). Surgeons with higher UKA usage had lower UKA revision rates, but higher TKA revision rates. The lowest TKA and combined UKA + TKA revision rates were observed for surgeons performing 1–5% UKA, compared to the highest TKA and UKA + TKA revision rates which were seen for surgeons using > 30% UKA (*p* < 0.001 TKA; *p* < 0.001 UKA + TKA). No clinically important differences in UKA + TKA OKS scores were seen between UKA usage groups at 6 months, 5 years, or 10 years.

**Conclusion:**

Surgeons with higher medial UKA usage have lower UKA revision rates; however, this comes at the cost of a higher combined UKA + TKA revision rate that is proportionate to the UKA usage. There was no difference in TKA + UKA OKS scores between UKA usage groups. A small increase in TKA revision rate was observed for high-volume UKA users (> 30%), when compared to other UKA usage clusters. A significant decrease in UKA revision rate observed in high-volume UKA surgeons offsets the slight increase in TKA revision rate, suggesting that UKA should be performed by specialist UKA surgeons.

**Level of evidence:**

III, Retrospective therapeutic study.

## Introduction

Patients with isolated medial compartment knee osteoarthritis (OA) can be treated surgically with a joint preserving high tibial osteotomy (HTO), or arthroplasty, either a medial unicompartmental knee arthroplasty (UKA) or total knee arthroplasty (TKA) [27]. In clinical practice, TKA is by far the more commonly used procedure, followed by UKA [2, 16].

If a patient undergoing arthroplasty meets the criteria for medial UKA [8], the surgeon and the patient need to decide if the faster recovery and lower perioperative morbidity after UKA are worth the cost of a higher revision rate compared to TKA [18, 30]. According to a 2009 eligibility study, the proportion of knee arthroplasty patients suitable for UKA is as high as 47.6% [29]. However, mean UKA usage worldwide remains around 10–15% of knee arthroplasty [16]. Since the main drawback of UKA is the higher revision rate, mitigation of the revision rate is a strong research focus [3, 9, 19, 20, 22]. Surgeons with a higher UKA usage are known to have lower UKA revision rates [9]. This has led to recommendations such as a ‘minimum’ surgeon UKA usage of 20% and an ‘optimum’ usage of 40–60% to minimize the risk of revision [19]. This increase in usage is supported by studies reporting similar clinical outcomes when using ‘extended’ UKA indications [8], accepting higher grade patellofemoral chondral change [13], or ignoring BMI [21].

However, as most surgeons have limited scope to control the casemix within their practice, to expand their UKA usage, they will need to proportionally reduce their usage of TKA. The impact of this decrease in TKA usage on TKA revision rate, and their overall combined UKA + TKA revision rate, remains unclear. As the ‘criteria’ for UKA tend to include higher functioning patients with preserved range of motion and minimal deformity who also do well following TKA, it is possible that maximizing UKA usage may adversely affect surgeons’ TKA results. Additionally, the ‘optimum’ UKA usage percentage to maximize overall patient-reported outcome measures (PROMS) remains unknown.

The aim of this study was to investigate how surgeon UKA usage influences the revision rate and PROMs of UKA, TKA, and combined UKA + TKA results.

It was hypothesized that higher UKA usage will decrease UKA revision rate. Based on the gap in the data on the influence of UKA usage on TKA and combined UKA + TKA results, a hypothesis was not possible for this research question.

## Methods

Data from the New Zealand National Joint Registry (NZJR) [24] for TKA and UKA during the time period January 2000 and December 2018 was analyzed. Included were patients undergoing any knee arthroplasty for a diagnosis of osteoarthritis, performed by surgeons with greater than 100 knee arthroplasty procedures recorded on the registry. This threshold was chosen to exclude knee arthroplasty surgeons who had not yet formed a practice pattern in regards to UKA usage. Lateral unicompartmental knee arthroplasties as well as patellofemoral knee arthroplasties were excluded, since these numbers procedures are rare [15, 28].

The cases were stratified by surgeon, by procedure and by year. Each surgeon’s UKA usage was calculated as the percentage of UKA from the combined number of UKA and TKA. Based on UKA usage, surgeons were then divided into six groups of UKA usage percentage: < 1.00%; 1.01–5.00%; 5.01–10.00%; 10.01–20.00%; 20.01–30.00%; > 30.01%. Due to a significantly higher revision rate for both UKA and TKA in patients under the age of 55 [6], a sub-analysis excluding patients under 55 at time of primary arthroplasty was also performed.

Outcome measures were all-cause revision rate of UKA, TKA, and the combined UKA + TKA revision rate per UKA usage group. Revision is defined in the NZJR as an open procedure where any component is removed, manipulated, exchanged, or implanted. Additionally, PROMS by UKA usage cluster using the Oxford Knee Score (OKS) were analyzed. The New Zealand Joint Registry collects PROMS routinely using the OKS. The Registry captures OKS after UKA and TKA at 6 months, 5 years and 10 years postoperatively, aiming for a 20% capture.

The New Zealand Joint Registry is funded from contributions from surgeons, Accident Compensation Corporation (ACC), the New Zealand government, and Southern Cross Hospital. It has an ongoing ethical approval obtained from Canterbury District Health Board in 1998. A separate ethics board approval was not necessary for the present study.

### Statistical analysis

Continuous data were presented with mean (± standard deviation) and compared with the independent *t* test. A two-step cluster analysis was performed to determine the UKA usage clusters. With the distribution of UKA percentages, and with one cluster set to a minimum of 20.00% UKA [19], the hierarchical cluster analysis determined six clusters. Then, the *K*-means cluster analysis was used to determine the final UKA usage clusters, with the final numbers rounded to the closest 0.00 decimals. The revision rates are reported using rate/100 component years to accommodate for the different observed periods [25]. The survivorship analyses were performed using the Kaplan–Meier analyses with revision as the event. Patients were censored either at the date of death or at the end of the follow-up period, if the event has not occurred. Comparison between the clusters was performed using the Log rank (Mantel Cox) test. Due to a difference in patient demographics between the clusters, age and gender were entered into the Cox regression analysis to generate adjusted hazard ratios for cluster comparisons. Patient-reported outcomes were compared using ANOVA, and if significant effects were identified pairwise comparisons were undertaken using independent samples *t* test. Due to the data availability as determined by the registry database size, a formal power analysis was not performed. Statistical significance was set at *p* < 0.05. SPSS 24.0 (IBM, Armonk, NY, US) was used for statistical analyses.

## Results

**Fig. 1:**
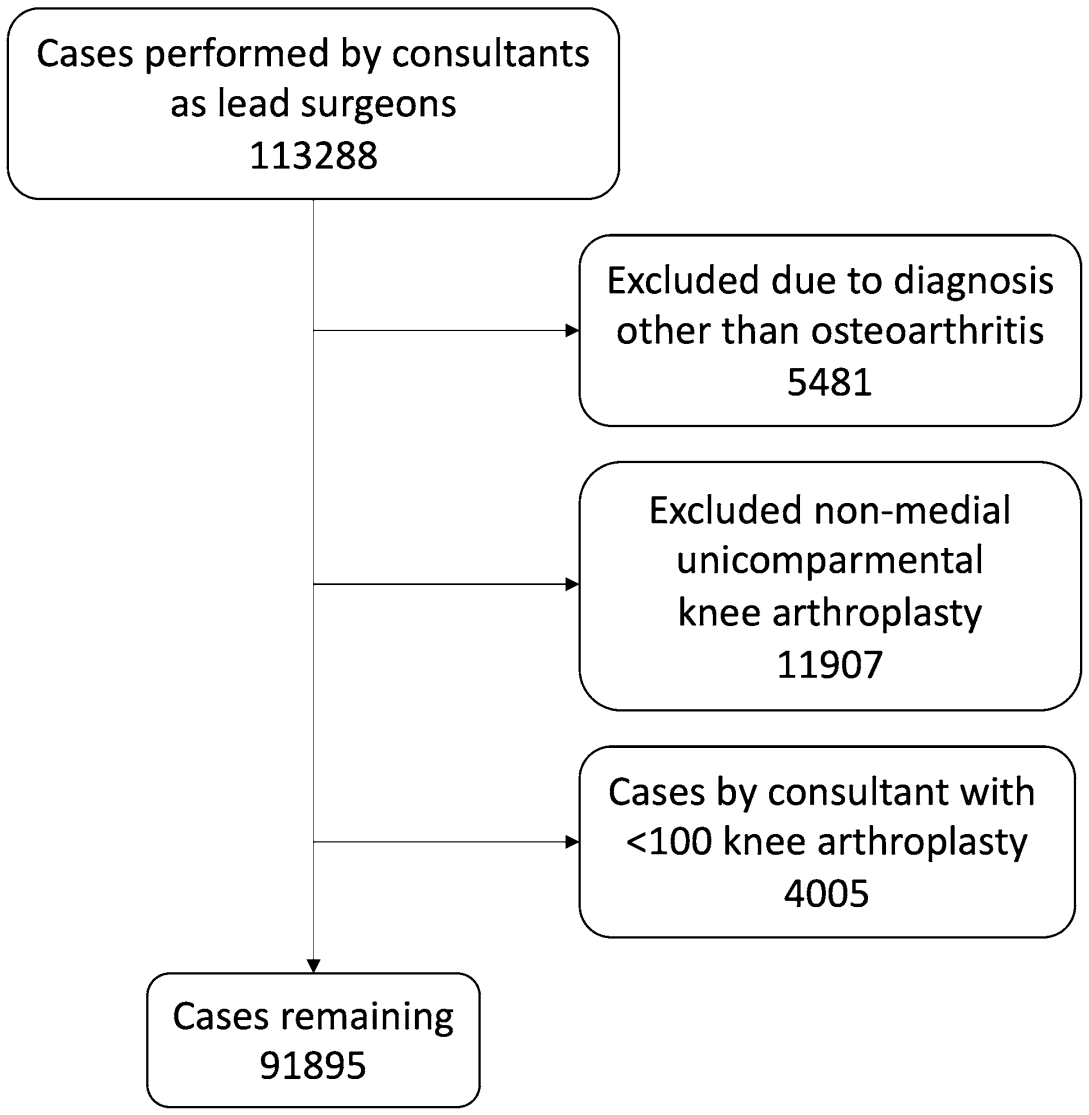
Flowchart of patient inclusion

In the study period, after application of inclusion and exclusion criteria, 216 consultants performed 91,895 knee arthroplasty procedures (Fig. 1). UKA was used in 9.0% of cases, 8271 procedures. An ‘average’ consultant performed 55 UKA and 425 TKA during a median of 14 years [3–18 years] of activity in the registry; therefore, averaging 4 UKA and 31 TKA per year. Casemix did differ between surgeon UKA usage groups, with higher UKA percentage surgeons operating on more males and younger patients (*p* < 0.001, Table 1.)

**Table 1:** Age and gender distribution between the clusters

Cluster UKA Usage	Number	Gender		Total	Age group				Total
	of surgeons	F	M		< 55	55–64	65–74	≥ 75	
0–1%									
*n*	85	15,063	14,508	29,571	2146	8090	11,680	7655	29,571
%		50.9	49.1		7.3	27.4	39.5	25.9	
1–5%									
*n*	35	8615	8165	16,780	1181	5009	6516	4074	16,780
%		51.3	48.7		7.0	29.9	38.8	24.3	
5–10%									
*n*	28	6614	6402	13,016	1165	3746	4910	3195	13,016
%		50.8	49.2		9.0	28.8	37.7	24.5	
10–20%									
*n*	40	9239	9214	18,453	1640	5289	6887	4637	18,453
%		50.1	49.9		8.9	28.7	37.3	25.1	
20–30%									
*n*	13	3225	3525	6750	558	1991	2502	1699	6750
%		47.8	52.2		8.3	29.5	37.1	25.2	
> 30%									
*n*	15	3456	3869	7325	658	2219	2747	1701	7325
%		47.2	52.8		9.0	30.3	37.5	23.2	
Total									
*n*	216	46,212	45,683	91,895	7348	26,344	35,242	22,961	91,895
%		50.3	49.7		8.0	28.7	38.4	25.0	

The lowest overall revision rate for TKA was observed in the 1–5% UKA usage group (0.40 per 100 component years (cy), 95% CI 0.64–0.44) and the highest in clusters 20–30% UKA and < 30% UKA (both 0.59 per 100 cy, 95% CI 0.51–0.67, Table 2). The lowest revision rate for UKA was observed in the > 30% UKA, of 1.0 per 100 cy (95% CI 0.86–1.14). Overall, the lowest combined UKA + TKA revision rate was seen in surgeons with a UKA usage of 1–5% (0.44 per 100 cy, 95% CI 0.4–0.48).

**Table 2:** Revision rates/100-component-years per cluster

Cluster UKA usage	Procedures	Component years	Revised	Rate/100-component-years	Lower 95% CI	Upper 95% CI
0–1%						
TKA	29,528	190,829.1	917	0.48	0.45	0.51
UKA	43	280.1	10	3.57	1.71	6.56
Total	29,571	191,109.3	927	0.49	0.45	0.52
1–5%						
TKA	16,438	100,829.5	406	0.40	0.36	0.44
UKA	342	2417.8	51	2.11	1.55	2.75
Total	16,780	103,247.3	457	0.44	0.40	0.48
5–10%						
TKA	12,118	82,421.1	404	0.49	0.44	0.54
UKA	898	7354.5	107	1.45	1.19	1.75
Total	13,016	89,775.6	511	0.57	0.52	0.62
10–20%						
TKA	15,895	107,448.0	493	0.46	0.42	0.50
UKA	2558	17,277.5	210	1.22	1.05	1.39
Total	18,453	124,725.6	703	0.56	0.52	0.61
20–30%						
TKA	5199	35,375.4	207	0.59	0.51	0.67
UKA	1551	9919.5	133	1.34	1.12	1.58
Total	6750	45,294.9	340	0.75	0.67	0.83
> 30%						
TKA	4446	32,859.9	193	0.59	0.51	0.67
UKA	2879	19,459.4	194	1.00	0.86	1.14
Total	7325	52,319.3	387	0.74	0.67	0.82
Total						
TKA	83,624	549,763.1	2620	0.48	0.46	0.50
UKA	8271	56,708.8	705	1.24	1.15	1.34
Total	91,895	606,471.9	3325	0.55	0.53	0.57

**Fig. 2:**
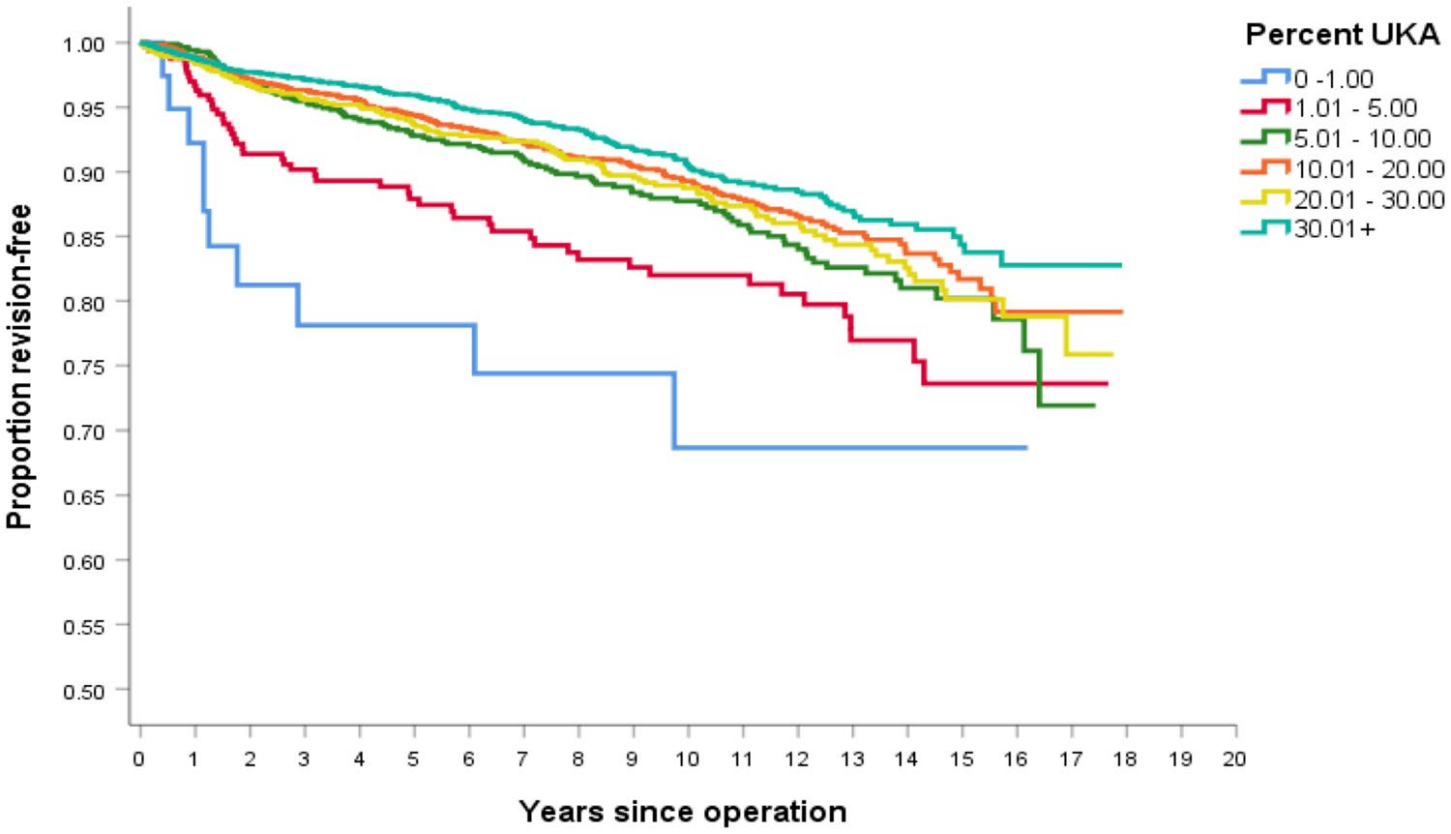
Kaplan–Meier survivorship curve of only UKA survivorship, for each of the six UKA clusters, based on UKA usage

Similar findings were observed when analyzing using Kaplan–Meier survivorship. The highest UKA survivorship was observed in > 30% UKA and the lowest in < 1% UKA, Fig. 2. The higher survivorship observed in surgeons with UKA usage > 30% was statistically significant compared to all clusters except UKA usage 10–20% (Table 3).

**Table 3:** Log rank (Mantel–Cox) comparison of UKA survivorship between the clusters. with *p* values reported

Cluster	0–1%	1–5%	5–10%	10–20%	20–30%	> 30%
0–1%		0.140	0.004	0.001	0.002	0.000
1–5%	0.140		0.029	0.000	0.005	0.000
5–10%	0.004	0.029		0.156	0.503	0.003
10–20%	0.001	0.000	0.156		0.436	0.056
20–30%	0.002	0.005	0.503	0.436		0.014
> 30%	0.000	0.000	0.003	0.056	0.014	

**Fig. 3:**
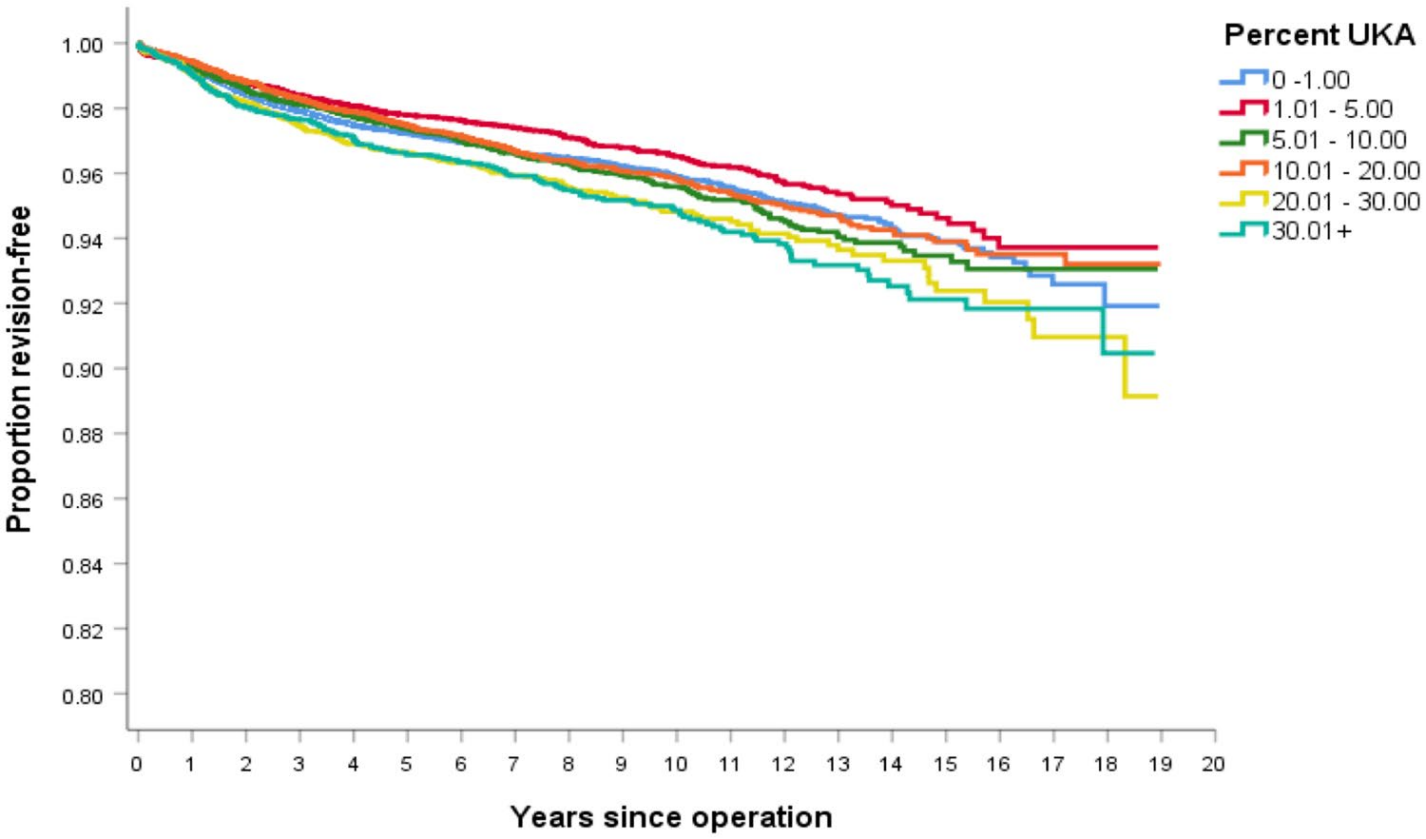
Kaplan–Meier survivorship curve of only TKA survivorship, for each of the six UKA clusters, based on UKA usage

When only TKA outcomes are analyzed, the lowest TKA revision rate was observed in the 1–5% UKA usage cluster, Fig. 3. Surgeons with the highest UKA usage observed a significant decrease in TKA survivorship, when compared to lower volume UKA users, Table 4. When controlling for age and gender, and excluding patients < 55 undergoing a TKA, the lowest hazard ratio for TKA revision remained the 1–5% UKA usage cluster, Table 5.

**Table 4:** Log rank (Mantel–Cox) comparison of TKA survivorship between the clusters, with *p* values reported

Cluster	0–1%	1–5%	5–10%	10–20%	20–30%	> 30%
0–1%		0.002	0.610	0.498	0.007	0.004
1–5%	0.002		0.002	0.029	0.000	0.000
5–10%	0.610	0.002		0.334	0.038	0.026
10–20%	0.498	0.029	0.334		0.003	0.002
20–30%	0.007	0.000	0.038	0.003		0.858
> 30%	0.004	0.000	0.026	0.002	0.858

**Table 5:** Cox regression analysis of TKA revision rates between clusters, controlled for age and gender, excluding patients < 55

Cluster	Wald	*p* value	Hazard ratio	95% CI for hazard ratio
0–1%	5.454	0.020	0.817	0.690–0.998
1–5%	15.366	< 0.001	0.690	0.573–0.830
5–10%	2.646	0.104	0.856	0.711–1.032
10–20%	7.701	0.006	0.772	0.644–0.927
20–30%	0.010	0.922	0.989	0.798–1.226

Cluster were tested against the cluster > 30%

**Fig. 4:**
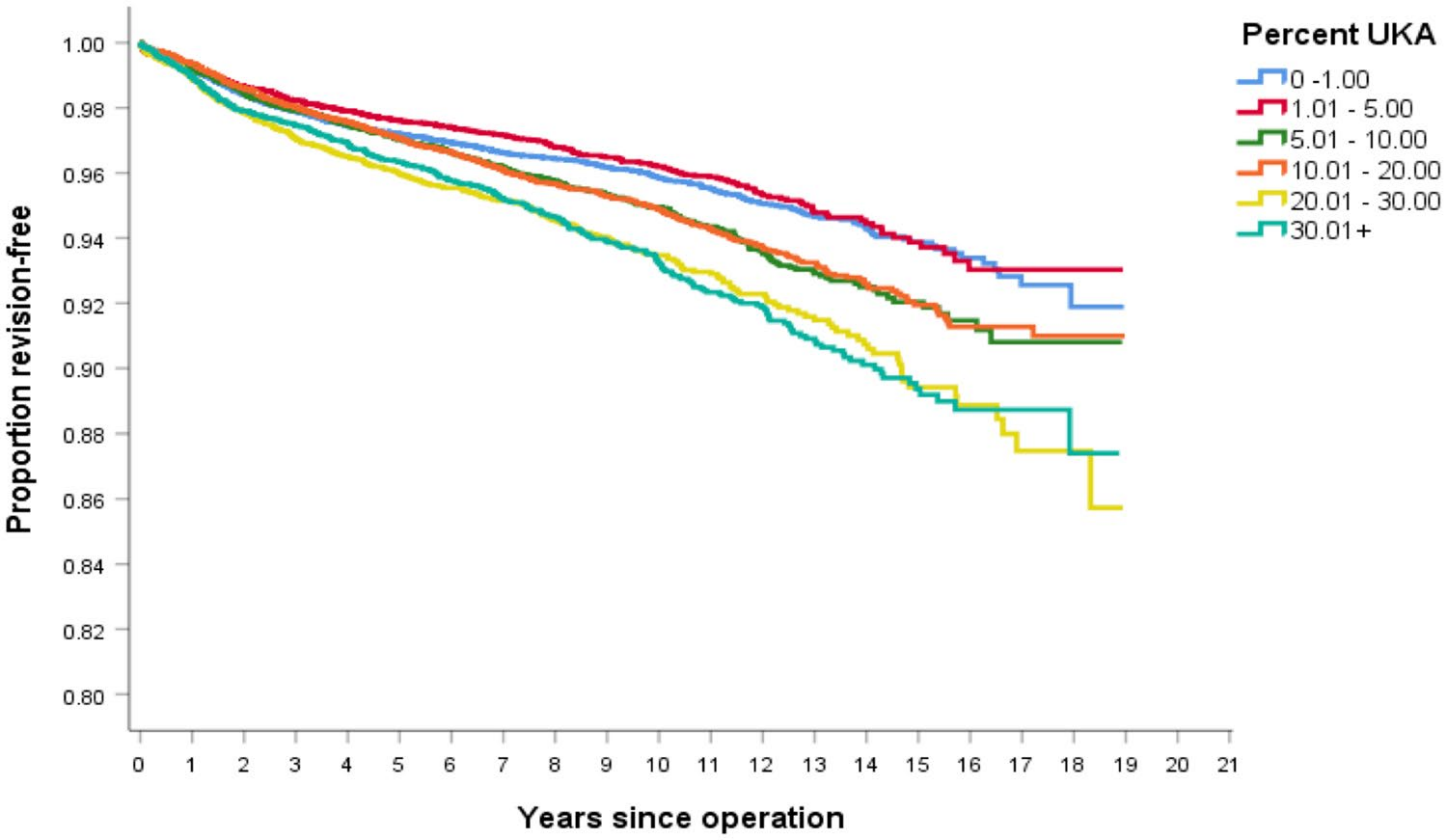
Kaplan–Meier survivorship curve of UKA + TKA survivorship, for each of the six UKA clusters, based on UKA usage

With increased usage of UKA, overall combined UKA + TKA survivorship decreases (Fig. 4.) There was no statistically significant difference in survivorship when compared to the cluster closest on the curve (Table 6).

**Table 6:** Log rank (Mantel–Cox) comparison of combined UKA/TKA survivorship between the clusters with *p* values reported

Cluster	< 1%	1–5%	5–10%	10–20%	20–30%	> 30%
< 1%		0.077	0.002	0.002	0.000	0.000
1–5%	0.077		0.000	0.000	0.000	0.000
5–10%	0.002	0.000		0.851	0.000	0.000
10–20%	0.002	0.000	0.851		0.000	0.000
20–30%	0.000	0.000	0.000	0.000		0.912
> 30%	0.000	0.000	0.000	0.000	0.912	

When analyzing PROMS, Table 7, the overall mean OKS was higher at 6 months (*p* < 0.001) in surgeons with UKA usage > 30% than in all other groups, although the absolute difference was not clinically relevant, ranging from 0.7 to 1.6 OKS points without a clear trend between groups. At 5 years and 10 years, there was little to no difference in overall mean OKS between UKA usage groups, Table 7.

**Table 7:** Oxford knee score analysis of combined UKA/TKA survivorship

Cluster	*N*	Mean (± SD)	*p* values Vs				
			1–5%	5–10%	10–20%	20%–30%	> 30%
6 months Oxford score							
0–1%	7366	37.4 (± 8.1)	< 0.001	< 0.001	< 0.001	0.226	< 0.001
1–5%	4697	38.5 (± 7.5)		0.295	0.169	< 0.001	0.002
5–10%	3917	38.3 (± 7.7)			0.848	0.001	< 0.001
10–20%	6270	38.3 (± 7.8)				0.001	< 0.001
20–30%	2681	37.7 (± 8.2)					< 0.001
> 30	3433	39.0 (± 7.5)					
Total	28,364	38.1 (± 7.9)					
5 years Oxford score							
0–1%	3018	40.5 (± 7.7)	0.027	0.025	0.189	0.597	0.019
1–5%	1745	41.0 (± 7.3)		0.968	0.313	0.030	0.803
5–10%	1719	41.0 (± 7.2)			0.294	0.028	0.833
10–20%	2590	40.8 (± 7.5)				0.143	0.222
20–30%	1004	40.4 (± 7.5)					0.021
> 30	1438	41.1 (± 7.3)					
Total	11,514	40.8 (± 7.5)					
10 years Oxford score							
0–1%	1428	40.0 (± 8.0)	0.595	0.638	0.294	0.698	0.117
1–5%	894	40.2 (± 8.0)		0.961	0.148	0.434	0.330
5–10%	859	40.2 (± 8.0)			0.168	0.463	0.312
10–20%	1318	39.7 (± 8.6)				0.673	0.016
20–30%	581	39.9 (± 7.9)					0.104
> 30	731	40.6 (± 7.6)					
Total	5811	40.1 (± 8.1)					

## Discussion

The most important finding of the present study is that high UKA usage is associated with a higher TKA revision rate, without an overall increase in OKS. The usage of UKA varies widely amongst knee arthroplasty surgeons, reflecting differing interpretations of the balance between advantages of UKA, such as a faster recovery and lower perioperative morbidity, versus a higher revision rate when compared to TKA [18, 30]. Similar to previous studies, we found surgeons with higher UKA usage achieve lower UKA revision rates.

Data from the NZJR in 2006 provided one of the first reports linking higher UKA usage with lower UKA revision rates [10], and since then a number of publications have attempted to quantify the ‘optimal’ usage of UKA. Baker et al. analyzed the UK National Joint registry data between 2003 and 2010, categorizing surgeons and centers based on the total number of UKAs performed, ranging from < 25 to > 200 [3]. Improved UKA survivorship was seen in both high-volume centers and high-volume surgeons. The authors concluded that surgeons undertaking UKA should perform at least 13 UKAs per year. Similarly, Liddle et al. analyzed the UK National Joint registry using fractional polynomials, that were then fitted using locally weighted scatterplot smoothing [19]. There was a steep drop of the revision rate once 20% UKA usage was reached, with the lowest area of revision percentage observed with UKA usage of between 40 and 60%. The authors concluded that, to reduce the revision risk significantly, the minimum UKA usage was 20%, with ‘optimum’ usage is 40–60%. Such studies exclusively analyze UKA outcomes. In contrast, the findings of the present study support that surgeons with high UKA usage will achieve improved UKA survivorship, their combined UKA + TKA survivorship remains lower than in surgeons with lower UKA usage percentages.

Surgeons aiming to increase their volume of UKA need to either alter their referral casemix to see more patients suitable for UKA, or broaden their indications for UKA [22]. A 2009 analysis suggested 48% of patients undergoing knee arthroplasty are potentially suitable for a UKA [29]. The findings of the present study suggest that such a high UKA percentage usage will lead to a higher overall TKA + UKA revision rate.

In evaluating combined UKA + TKA revision rates, the present study assumes that a revision from a UKA is similar to a revision from a primary TKA. Many surgeons prefer to use a UKA in younger patients undergoing arthroplasty, on the basis of such patients being more likely to require a revision procedure during their lifetime. The assumption is that the first revision will be more technically straightforward and have an improved outcome if it is from a primary UKA than from a primary TKA. However, a number of studies have suggested that the outcome of revision UKA is more comparable to that of a revision TKA than to a primary TKA [17, 26] and that revision rates of a UKA revised to a TKA are high, rendering a UKA not an intermediate procedure [12]. Furthermore, the vast majority of patients undergoing both TKA and UKA will not require a revision procedure during their lifetime, so any revision procedure can be considered an undesirable outcome, even in younger patients. The 18-year revision rate in patients < 55 undergoing primary UKA is 40.6%, compared to 17.8% for primary TKA patients in the same age group [23]. The indication for arthroplasty in a young patient should, therefore, be very carefully evaluated.

When analyzing combined UKA + TKA PROMS, an advantage in combined outcomes in the higher UKA usage (> 30%) group at 6 months, but not at 5 years or 10 years postoperatively was observed. The absolute difference of 0.7–1.4 points on the OKS is below the minimally important difference for this questionnaire, previously reported at 5 points [7]. These findings support those of previous RCTs finding similar functional outcomes for UKA and TKA [1, 4]. The most updated systematic review and meta-analysis comparing UKA and TKA patient specific outcomes and revision found no difference in pain, with functional PROMS higher for UKA than for TKA in both non-trial groups [30]. However, some TKA patients in non-trial groups may not be suitable for UKA, due to a more extensive disease, greater deformity or a significantly reduced range of motion. There may also be a difference in functional outcome in high demand patients that is not identified when using PROMS such as OKS due to the ceiling effect [14].

### Limitations

There are a number of limitations in the present study. Higher usage UKA surgeons might have differing referral patterns and subsequent casemix, receiving tertiary referrals for more patients meeting UKA criteria, although there is also evidence against this [11]. Evidence for this is seen in the higher percentage of younger, male patients in the high UKA usage groups in the present study. However, the difference in survivorship between usage groups remained when controlling for age and gender. Furthermore, absolute differences in OKS remained small, despite the presumably higher functioning patients seen by this group. The aimed capture rate of 20% may not represent the cohort adequately. However, a large number of patients that have not been captured should have a significantly higher or lower OKS to significantly alter the scores. Furthermore, the number of patients at 10-year follow-up is lower than for shorter follow-ups; over time there might be a difference with increased power. Second, the NZJR lacks pre-operative OKS scores and X-rays to accurately classify patients. Therefore, the specific indications of each surgeon for UKA, TKA, or other management options, such as osteotomy or non-operative therapies, remain unknown. However, by analyzing outcomes for a large number of knee surgeons across a broad time period, this study provides an overall picture of patients undergoing knee arthroplasty in New Zealand. Third, the surgeon UKA usage was grouped as a percentage of overall arthroplasty volume, which does not account for changing usage patterns during careers or an annual volume. Some studies report improved UKA outcomes with a higher absolute number of UKAs performed per year [5, 9], rather than a percentage UKA + TKA, which were not controlled in this study. However, surgeons perform more UKA cannot easily change the number of knee arthroplasty patients in their practice, rather they adjust their clinical UKA ‘threshold’ which is better represented by a percentage UKA usage than a volume per year. Finally, other important outcomes that may differ between UKA and TKA were not captured by this study, such as length of hospital stay, range of motion, and perioperative complications. However, revision rate and patient-reported outcomes such as the OKS remain important considerations in the decision between UKA and TKA for surgeons.

## Conclusions

Surgeons with higher medial UKA usage have lower UKA revision rates; however, this comes at the cost of a higher combined UKA + TKA revision rate that is proportionate to the UKA usage. There was no difference in TKA + UKA OKS scores between UKA usage groups. A small increase in TKA revision rate was observed for high-volume UKA users (> 30%), when compared to other UKA usage clusters.

A significant decrease in UKA revision rate observed in high-volume UKA surgeons offsets the slight increase in TKA revision rate, suggesting that UKA should be performed by specialist UKA surgeons.
